# The paradox of healing

**DOI:** 10.12669/pjms.40.11.11015

**Published:** 2024-12

**Authors:** Sheraz Jamal Khan, Mehwash Iftikhar

**Affiliations:** 1 Prof. Dr. Sheraz Jamal Khan Professor of Medicine, Ex-Associate Dean, Head, Department of Medicine, Postgraduate Studies, Hayat Abad Medical Complex, Peshawar, Pakistan; 2 Dr. Mehwash Iftikhar Specialty Registrar, Hayat Abad Medical Complex, Peshawar, Pakistan

In the tale of human suffering, few bear a more ironic burden than doctors. While society holds them in the highest regard, their lives are filled with contradictions. Every meal they earn is linked to pain, and every pay-check serves as a reminder of human suffering. Doctors do not simply witness suffering; they are deeply entangled in it, walking a delicate line between the desire to heal and the harsh reality of mortality.

Where writers wrestle with deep existential questions—like the nature of suffering or the hope for redemption—doctors face the relentless routine of patient care. Human lives are often reduced to problems to be solved, rather than mysteries to be pondered. No medical student flipping through Gray’s Anatomy^1^ envisions a future in which their livelihood is bound to constant encounters with illness and death. No one dreams of a profession where every penny they earn is linked to someone else’s sorrow. And yet, here they stand, caught in what seems like a Faustian pact, exchanging years of study and sleepless nights for societal respect and the crushing weight of responsibility.

It’s akin to the tragic irony of Shakespeare’s Macbeth, where he cries, “Out, out brief candle”,^2^ as his life unravels. Today’s physician might mutter, “Out, out cellphone’s battery,” knowing it heralds yet another call to action. The profession promises to “do no harm,” as enshrined in the Hippocratic Oath,^3^ but it remains silent about the toll it exacts on the doctor’s health—sleep deprivation, strained relationships, and a personal well-being constantly at risk.

Like Kafka’s characters, trapped in endless bureaucratic systems,^4^ doctors are engulfed in paperwork, regulations, and the limitations of modern healthcare. The pursuit of healing can feel as futile as Sisyphus’ eternal struggle to push his boulder uphill, only to watch it roll back down.^5^ And yet, as Camus suggested, we must imagine Sisyphus happy.^6^ Perhaps doctors too must find peace amidst the absurdity, driven by a profound sense of purpose—or at the very least, the ability to laugh through the chaos.

The surgeon, scalpel in hand, lives with a sword of Damocles perpetually hanging overhead.^7^ Each incision serves a dual purpose: to heal the patient and to secure the surgeon’s own livelihood. Every successful operation is both a victory and a means to sustain their life. This bitter irony is one Shakespeare would have appreciated—a modern twist on The Merchant of Venice, where instead of a pound of flesh, we exchange illnesses and surgeries for financial security.^8^

The tension between healing and profit has long been a target of satire, as seen in Molière’s The Imaginary Invalid,^9^ where doctors exploit imaginary diseases for monetary gain. While presented humorously, today’s medical world sometimes mirrors this, with physicians half-joking, “I make a living from diseases I wish didn’t exist.” Yet the laughter is tinged with sadness, as the truth cuts too deeply to ignore.

Franz Kafka once remarked, “A book must be the axe for the frozen sea within us”.^10^ For doctors, that frozen sea represents the emotional distance they cultivate to survive in a world soaked in suffering. Without it, they risk being consumed by the unending tide of illness and death. Yet too much detachment leaves them cold and mechanical, treating conditions instead of patients. Doctors must walk a fine line, balancing empathy and efficiency, knowing that with each life saved, more suffering awaits.

In many ways, doctors resemble Camus’ Meursault in The Stranger^11^—coolly navigating a world indifferent to human suffering, performing their duties as the world around them disintegrates. Physicians must come to terms with an indifferent universe, where illness, recovery, life, and death cycle endlessly. Ultimately, their profession, like life itself, is full of paradoxes. Healing is noble, but it is also a means to a livelihood. Doctors traverse this moral labyrinth like characters in Kafka’s works—laughing at the absurdity, knowing that in a world devoid of suffering, they would have no paycheck. And yet, as with all great literature, there is a strange comfort in knowing these struggles are timeless, stretching from Kafka to Camus, from Molière to Shakespeare.
